# Identify-Isolate-Inform: A Modified Tool for Initial Detection and Management of Middle East Respiratory Syndrome Patients in the Emergency Department

**DOI:** 10.5811/westjem.2015.7.27915

**Published:** 2015-10-20

**Authors:** Kristi L. Koenig

**Affiliations:** University of California, Irvine, Department of Emergency Medicine, Irvine, California. University of California, Irvine, Center for Disaster Medical Sciences, Irvine, California

## Abstract

Middle East respiratory syndrome (MERS) is a novel infectious disease caused by a coronavirus (MERS-CoV) first reported in Saudi Arabia in September 2012. MERS later spread to other countries in the Arabian Peninsula, followed by an outbreak in South Korea in 2015. At least 26 countries have reported MERS cases, and these numbers may increase over time. Due to international travel opportunities, all countries are at risk of imported cases of MERS, even if outbreaks do not spread globally. Therefore, it is essential for emergency department (ED) personnel to be able to rapidly assess MERS risk and take immediate actions if indicated. The Identify-Isolate-Inform (3I) tool, originally conceived for initial detection and management of Ebola virus disease patients in the ED and later adjusted for measles, can be adapted for real-time use for any emerging infectious disease. This paper reports a modification of the 3I tool for use in initial detection and management of patients under investigation for MERS. Following an assessment of epidemiologic risk factors, including travel to countries with current MERS transmission and contact with patients with confirmed MERS within 14 days, patients are risk stratified by type of exposure coupled with symptoms of fever and respiratory illness. If criteria are met, patients must be immediately placed into airborne infection isolation (or a private room until this type of isolation is available) and the emergency practitioner must alert the hospital infection prevention and control team and the local public health department. The 3I tool will facilitate rapid categorization and triggering of appropriate time-sensitive actions for patients presenting to the ED at risk for MERS.

## INTRODUCTION

**Figure 1:**
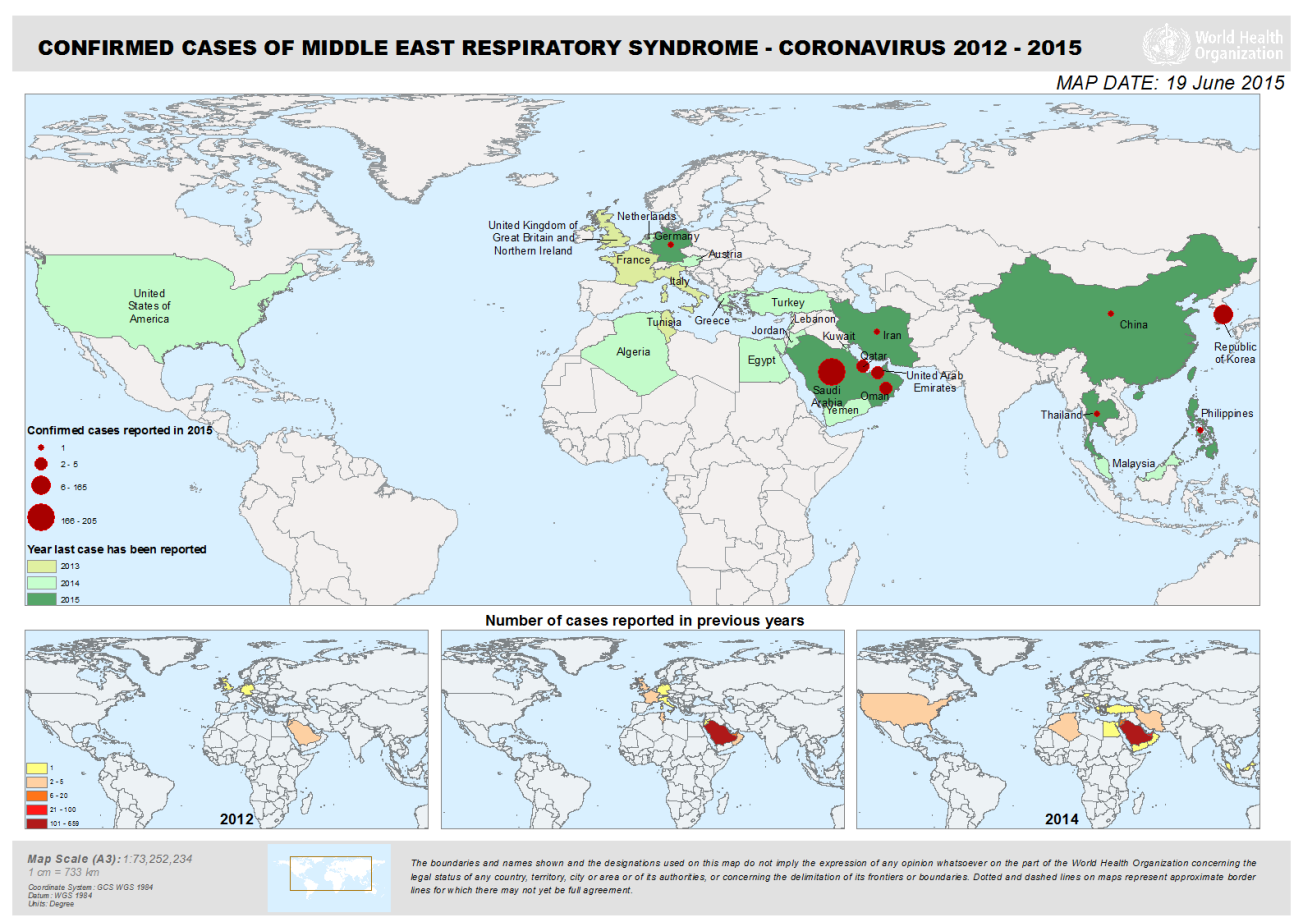
Countries with confirmed MERS cases as of June 19, 2015. *MERS*, Middle East respiratory syndrome

Middle East respiratory syndrome coronavirus (MERS-CoV) is a new respiratory virus that was first reported in Saudi Arabia in September 2012. Health officials later determined that the first known cases of MERS occurred in Jordan in April 2012. The outbreak extended to other countries in the Arabian Peninsula followed by another outbreak in Korea in May 2015. As of June 18, 2015, when Thailand described a case in a traveler returning from Oman, at least 26 countries have reported MERS cases (Figure 1). There have been at least 1,338 persons infected and 484 deaths (36% mortality) as of June 20, 2015.^1^ Transmission of the virus has occurred in healthcare facilities in Saudi Arabia and Korea, raising concerns such as those seen during the prior severe acute respiratory syndrome (SARS) outbreak first recognized in February 2003. Outside of the isolated outbreak regions, the vast majority of MERS cases have been detected in travelers returning from the Middle East. The list of affected countries may change over time, which can affect the exposure criteria for identifying suspected cases.

In May 2014, the Centers for Disease Control and Prevention (CDC) confirmed two unlinked imported cases of MERS in the U.S. states of Indiana and Florida. Both patients were believed to have been infected in Saudi Arabia where they worked as healthcare providers. They both required hospitalization and fully recovered.

While previously healthy individuals with mild illness may be asymptomatic, MERS typically presents with fever and symptoms of a respiratory illness or acute respiratory distress syndrome (ARDS) in severe cases. Suspect patients must be immediately isolated with airborne precautions concurrent with work up and laboratory confirmation of disease. In addition, emergency physicians must promptly inform both hospital infection control and the local health department of suspected MERS cases.

As with all emerging infectious diseases,^2^ healthcare workers must keep up to date with information about how to detect and manage MERS. Using the Identify-Isolate-Inform (3I) tool, emergency physicians will be better prepared to detect and manage MERS patients presenting to the emergency department (ED). Following a brief review of MERS, this paper describes the adaptation of the 3I tool, initially developed for Ebola virus disease,^3^–^4^ and modified for measles^5^ for use in the initial detection and management of potential MERS patients in the ED. While the MERS 3I tool is designed for use in EDs affiliated with an inpatient facility, it can be used in outpatient settings such as urgent care clinics, physicians’ offices and prehospital environments with minor modifications, e.g. if airborne isolation is not immediately available. The model presented is consistent with CDC guidelines for the management of suspected MERS patients.^6^

### Clinical Presentation

#### Signs and Symptoms

MERS-CoV infection presents as a nonspecific acute respiratory illness. Patients typically have fever, cough and shortness of breath. Gastrointestinal symptoms can include nausea, vomiting and diarrhea. Severely ill patients may develop pneumonia, ARDS and renal failure. Mortality is reported to be about 30–40% and occurs more commonly in people with underlying medical conditions. Some patients may be infected and fully recover, having either no symptoms or a mild respiratory illness without progression to severe disease. This cohort, however, is less likely to present to the ED.

The incubation period for MERS ranges from 2–14 days, typically about 5–6 days. This is the reason for screening for risk factors within 14 days prior to symptom onset. Comorbidities, such as diabetes, cancer, and chronic heart, lung, and kidney disease, portend a greater risk of contracting MERS and of progressing to more severe illness.

#### Transmission

While it is unclear exactly how MERS is contracted, it is likely to spread via an infected person’s respiratory secretions like other coronaviruses. To date, there has not been widespread sustained community human-to-human transmission. It appears that close contact with an infected person is necessary for disease transmission. Close contact is defined as encountering a patient without appropriate protective gear within six feet or being in a care room for prolonged periods or having direct exposure to infected secretions. Healthcare facilities have reported spread from person-to-person much more so than in communities, possibly when suboptimal infection control was practiced for patients with higher viral loads than those not hospitalized.^7^

**Figure 2:**
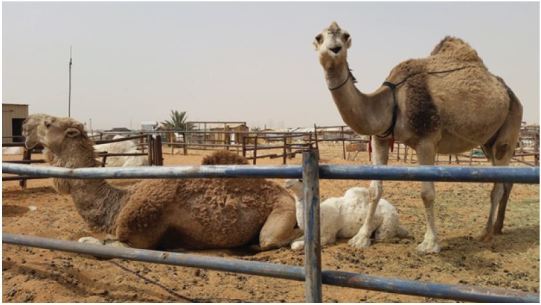
Photo from camel market outside Riyadh, Saudi Arabia where MERS-CoV(Middle East respiratory syndrome-Coronavirus) was first detected. Dromedary (one-hump) camels are strongly linked to having a role as a MERS-CoV reservoir and source of zoonotic transmission to humans.

Reported cases have been linked to countries in and near the Arabian Peninsula either for persons who live in, have traveled to, or have had contact with an infected person who had been in the region. MERS is a zoonotic virus that is transmitted from animals to humans. It is believed to have originated in bats and then to have been transmitted to camels sometime in the distant past. According to epidemiologic and surveillance data, there is a strong likelihood that dromedary (one-hump) camels (Figure 2) serve as a reservoir for zoonotic transmission of the virus to humans.^8^ This has resulted in warnings to avoid close contact with camels and not drink raw camel milk or urine, or ingest raw camel meat.

#### People Who May Be at Increased Risk for MERS

In addition to persons who have had close contact with infected dromedary camels within 14 days before symptom onset, the following groups are at risk for contracting MERS:

- Travelers from the Arabian Peninsula (note that geographic regions of concern may change over time)
- Close contacts of an ill traveler from the Arabian Peninsula
- People who have been in a healthcare facility in the Republic of Korea
- Close contacts of a confirmed case of MERS

Elderly and immunocompromised patients are at higher risk of becoming infected with MERS than healthy hosts if they are exposed to the conditions described above.

#### Work-Up

MERS can be confirmed at a state or CDC laboratory via polymerase chain reaction (PCR) assays performed on respiratory samples. In addition, serum antibody titers can be measured for both acute infection as well as evidence of prior exposure and immunity. Serologic testing includes (1) enzyme-linked immunosorbent assay (ELISA) as a screening test, (2) immunofluorescent assay (IFA) for confirmation, and (3) neutralizing antibody assay as a definitive confirmatory test that takes longer to process.

#### Differential Diagnosis

As MERS presents initially with a non-specific influenza like illness, the differential diagnosis can include many other respiratory and gastrointestinal infections. The key action is to identify a potential exposure within 14 days prior to symptom onset at initial patient presentation so that MERS can be considered.

#### Treatment

Treatment for MERS is primarily supportive care. Hydration and antipyretics, such as in other viral illnesses, are the mainstays of therapy. If a secondary bacterial infection such as pneumonia develops, appropriate antibiotics are indicated. While drugs for treatment of severe acute respiratory syndrome such as beta interferons and protease inhibitors are reported to be under investigation,^9^ there are currently no approved specific treatments or vaccines for MERS.

#### Prevention

Prevention of MERS-CoV transmission involves avoiding exposure. Travelers to regions where MERS has been detected should avoid close contact with potentially infected persons or dromedary camels. Healthcare personnel must practice strict standard, contact, and airborne precautions while caring for patients under investigation (including symptomatic close contacts) as well as patients with probable or confirmed MERS infections. Laboratory workers and others collecting and handling specimens for potential MERS patients should adhere to the same guidelines. Adequate respiratory protection is particularly important when performing aerosolizing procedures.

#### Patient Disposition

Admission criteria for patients who are at risk for MERS are similar to those for any other patient. If patients do not meet medical criteria for hospitalization, they may be isolated at home during the evaluation period. Emergency physicians must notify local public health authorities so that appropriate monitoring and community protective measures can be instituted. Return precautions should include attention to any signs or symptoms of pneumonia, ARDS or renal failure.

### Identify-Isolate-Inform

**Figure 3:**
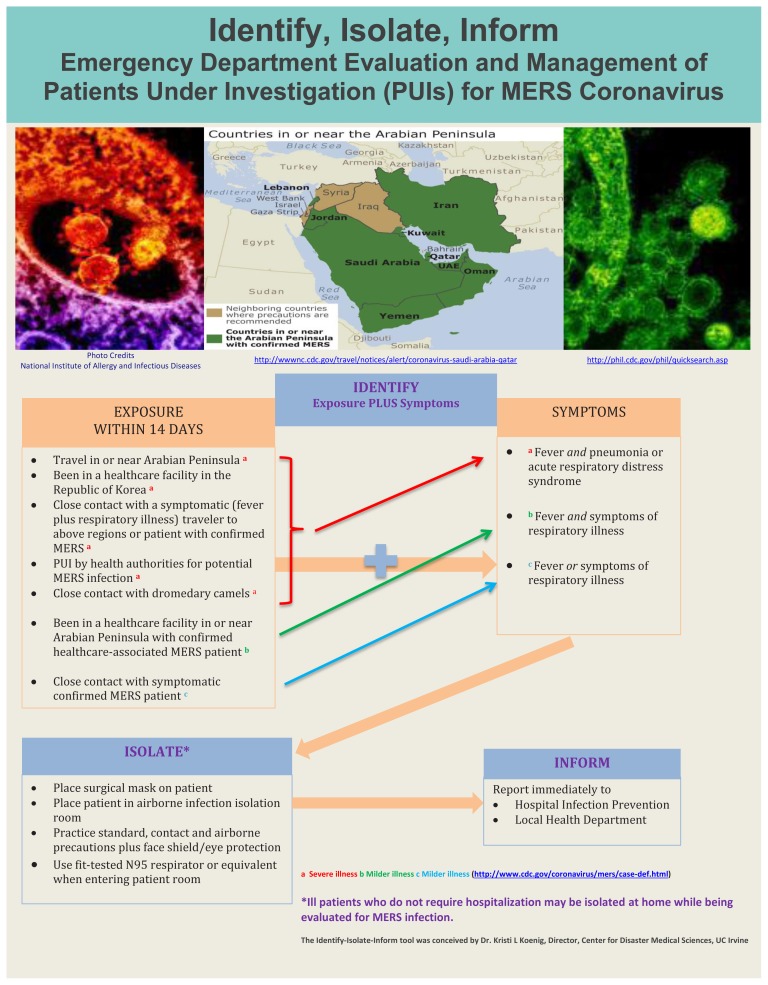
Koenig’s Identify-Isolate-Inform tool adapted for Middle East respiratory syndrome.

The Identify-Isolate-Inform tool initially developed for Ebola virus disease and subsequently adapted for measles can be modified for the ED evaluation and management of patients under investigation (PUI) for MERS (Figure 3). A PUI is a person who has both clinical features of MERS and an epidemiologic risk factor. The MERS tool could be accessed real-time on a triage nurse’s computer screen or printed as a poster for display in the triage area. The first step is to identify patients with a possible MERS-CoV exposure within 14 days before symptom onset. CDC and the World Health Organization (WHO) provide case definitions that are comprehensive,^10^,^11^ but do not lend themselves to use by frontline emergency personnel who must make rapid risk assessments. Therefore, the 3I tool provides a concise and simplified version of exposure types coupled with symptoms for both severe and milder illness.

If a patient is not identified as having an exposure risk coupled with symptoms, triage may proceed as usual. A caveat is that the Vital Sign Zero^12^ concept must be applied to all patients before direct patient contact is made to measure traditional vital signs. Vital Sign Zero refers to a mindset of first determining whether the patient may be a risk to expose or contaminate healthcare personnel prior to them having contact with the patient in order to measure traditional vital signs. By first assessing whether the patient is contaminated or contagious, the healthcare provider can don risk-appropriate personal protective equipment before continuing with a full evaluation.

For patients who have positive exposure plus symptom findings, the second step in the algorithm is to immediately “isolate.” A surgical mask should be placed on such patients and they should be directed to an airborne infection isolation room. (If airborne isolation is not available, the patient should be placed in a private room until transfer to an appropriate facility can be arranged.) Staff entering the room should adhere to standard, contact and airborne precautions. They should don appropriate PPE to include a fit-tested N95 respirator or equivalent, eye protection, gown and gloves. Isolated patients should have samples obtained urgently and sent to the local public health department laboratory for disease confirmation.

The final action of the tool is to “inform.” In addition to notifying the hospital infection prevention and control team, emergency physicians should promptly report suspected MERS patients to the local public health department at all times of the day or night. Additional stakeholders, including hospital leadership, occupational health, and the laboratory would need notification through established communications processes at the facility.

Patients who do not meet medical criteria for admission can be isolated at home during the evaluation phase. However, as MERS is a serious contagious disease, an assessment of the home environment must first be performed. The patient needs to be reliable and compliant with home isolation. The home environment needs to have adequate support to offer proper care, including the means for a rapid return for reevaluation if the patient’s condition deteriorates. Health department officials can assist with providing such patients with appropriate public health monitoring and measures to prevent infection transmission.

### Areas of Ambiguity

While the WHO uses the terminology MERS-CoV, they specifically suggest that the name should be avoided, stating that such nomenclature may have “unintended negative impacts by stigmatizing certain communities or economic sectors.”^13^ In addition, as has been the case for other emerging infection diseases such as the 2009 H1N1 pandemic, even purely science-based guidance from authoritative bodies is sometimes conflicting. For example, WHO recommends droplet precautions (surgical mask) unless an aerosolizing procedure is being performed, whereas the U.S. CDC endorses airborne precautions (N95 respirators or equivalent) for all circumstances. Even though there is no good evidence that the virus is transmitted by airborne routes (in the absence of aerosolizing procedures), some would argue that, to avoid transmission, it is better to be more conservative. However, this approach is not without downsides as, if the virus becomes more widespread, it could result in shortages of N95 respirators. Such shortages occurred during the 2009 H1N1 pandemic leading to concerns that respirators might be unavailable for patients with clear indications, such as those with tuberculosis. There is also a substantial cost both for purchasing and stockpiling and for training and fit testing for each new brand of N95 respirator if this approach is used.

Another challenging area is that of lack of standardization in case definitions. WHO and CDC information overlap but is not entirely the same. For example, CDC makes no mention of close contact with dromedary camels in the 14 days prior to symptom onset as a MERS risk factor.

As with all contagious infectious diseases, the question of when to use the public health tools of quarantine and isolation is critical.^14^,^15^ While it is clear that ill patients should be immediately isolated, the efficacy of the use of quarantine is more ambiguous. In general quarantine of asymptomatic patients is only beneficial in cases where the infected person is contagious prior to the onset of symptoms. For example, in the case of Ebola, other public health monitoring tools would make more scientific sense than quarantine as the disease becomes contagious only after symptom onset.^4^,^14^,^15^ As with other respiratory viruses, MERS may be contagious prior to symptom onset, but it does not seem to be easily transmissible from person to person. Furthermore, if it can be transmitted prior to symptom onset, it is unclear how many days prior. Given the current state of knowledge, avoidance of exposure, and, if exposed, implementation of public health monitoring measures other than quarantine are probably appropriate.

## CONCLUSION

MERS is an emerging infectious disease that is not yet fully understood in terms of mode of transmission and potential for widespread dissemination. As with any novel infection, it is important not only to identify and treat individual patients, but also to protect healthcare providers and the public health. The Identify-Isolate-Inform tool can be used real-time on the front lines to rapidly detect and manage patients at risk for MERS presenting to the ED. As with the similar 3I tools for Ebola and measles, it can be applied in any acute care setting such as clinics and prehospital environments. Use of the 3I tool will aid emergency physicians and other emergency personnel in performing rapid and appropriate screening for MERS.
